# Pironetin reacts covalently with cysteine-316 of α-tubulin to destabilize microtubule

**DOI:** 10.1038/ncomms12103

**Published:** 2016-06-30

**Authors:** Jianhong Yang, Yuxi Wang, Taijing Wang, Jian Jiang, Catherine H. Botting, Huanting Liu, Qiang Chen, Jinliang Yang, James H. Naismith, Xiaofeng Zhu, Lijuan Chen

**Affiliations:** 1 State Key Laboratory of Biotherapy and Cancer Center, West China Hospital of Sichuan University and Collaborative Innovation Center of Biotherapy and Cancer, Chengdu 610041, China; 2 College of Life Science, Sichuan University, Chengdu 610064, China; 3 Biomedical Sciences Research Complex, University of St. Andrews, St Andrews KY16 9ST, UK

## Abstract

Molecules that alter the normal dynamics of microtubule assembly and disassembly include many anticancer drugs in clinical use. So far all such therapeutics target β-tubulin, and structural biology has explained the basis of their action and permitted design of new drugs. However, by shifting the profile of β-tubulin isoforms, cancer cells become resistant to treatment. Compounds that bind to α-tubulin are less well characterized and unexploited. The natural product pironetin is known to bind to α-tubulin and is a potent inhibitor of microtubule polymerization. Previous reports had identified that pironetin reacts with lysine-352 residue however analogues designed on this model had much lower potency, which was difficult to explain, hindering further development. We report crystallographic and mass spectrometric data that reveal that pironetin forms a covalent bond to cysteine-316 in α-tubulin via a Michael addition reaction. These data provide a basis for the rational design of α-tubulin targeting chemotherapeutics.

## Abstract (web summary)

Microtubule assembly and disassembly is the target of many anticancer therapies, with β-tubulin the most-frequent target. Here, the authors used biochemical and biophysical techniques to demonstrate pironetin binds to α-tubulin and thereby inhibits microtubule polymerization providing a basis for the rational design of novel anticancer drugs.

## Graphical abstract

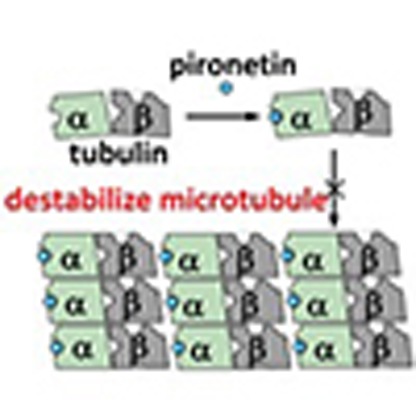

Microtubules act as the ‘skeleton of the cell', as well as guides and anchors for many cell events. The α, β-tubulin heterodimer reversibly assembles into protofilaments of microtubules (Supplementary Fig. 1a). Changes in this highly dynamic process are tightly controlled and associated with different stages of the cell cycle and replication^1^. The natural products vinblastine, colchicine, taxol, laulimalide and maytansine all bind to β-tubulin and interfere with the normal dynamic processes of tubulin: vinblastine, colchicine and maytansine inhibit polymerization of the tubulin heterodimer into microtubules^2^,^3^ while taxol and laulimalide promote polymerization and stabilize microtubules^2^,^4^. Structural biology has described in detail how these five natural compounds bind to and change the conformation of β-tubulin altering the dynamics between tubulins and microtubules^5^,^6^,^7^,^8^,^9^,^10^,^11^ (Supplementary Fig. 1b–e). The work has enabled the rational design of new generation of analogues with desirable properties although none are as yet in clinical use^12^,^13^,^14^. Taxol represents one of the most successful anticancer agents in current clinic use^2^,^4^. However, in patients undergoing therapy, the increased expression of β-tubulin isoforms is well known. These isoforms bind less strongly to taxol, resulting in drug-resistant cancers^2^,^4^,^15^.

Pironetin, an α/β unsaturated lactone originally isolated from fermentation broths of S*treptomyces* species, remains the only natural compound currently known to bind to α-tubulin^16^ (Fig. 1). Pironetin is a potent inhibitor of the cell cycle halting cells at M phase (IC_50_ in the range of 1.5–26 nM (refs 17, 18)) and at a dose of 6.3 mg kg^−1^ intravenous injection inhibits tumour growth in a mouse model of leukaemia^16^,^19^. Importantly, pironetin was found to be effective against cell lines resistant to other microtubule-targeted drugs and multidrug-resistant cells with *mdr1* gene expression^20^. *In vitro* and *in vivo* studies showed the compound acts by inhibiting the polymerization of tubulin and preventing assembly of the mitotic spindle^21^. The inhibition was discovered to be essentially irreversible under physiological conditions and this led to the prediction that the compound is a covalent modifier^22^. A detailed study involving site-directed mutagenesis, peptide sequencing, in part use of a biotinylated analogue of pironetin (Supplementary Fig. 2), cell biology and molecular modelling concluded that pironetin was covalently attached to Lys352 of α-tubulin^22^. The study explicitly ruled out pironetin binding to cysteine residues although from simple chemical consideration the more nucleophilic cysteine would be expected to be more reactive in a Michael-type addition to the lactone (nucleophilic addition to α/β unsaturated carbonyl) (Fig. 1). This study has formed the basis for the rational structure-based design of pironetin analogues^23^. Disappointingly, the pironetin analogues designed and synthesized on the Lys352 model showed much worse activity (often 1,000-fold worse) than the parent pironetin^24^,^25^. Further, rationalizing the structure–activity relationships of these compounds has not proven straightforward, which complicates design of a new generation of analogues^19^,^21^,^26^. The development of pironetin-based compounds as novel anticancer agents has stalled.

The attraction of an α-tubulin-binding microtubule-modifying agent is however very strong. A drug based on such a compound would almost certainly be insensitive to changes in expression of β-tubulin isoforms and therefore could help treat drug-resistant tumours. To provide a firmer basis for development of novel analogues, we have reinvestigated the molecular basis of pironetin activity. Our results do not support the previously published model of pironetin binding^22^. Rather structural biology and mass spectrometry studies using authentic pironetin (not the biotinylated analogue used previously^22^) show that pironetin is covalently bound by Cys316 of α-tubulin where it makes an extensive set of specific interactions with the protein. The data suggest a rational for the molecular basis of inhibition of microtubule polymerization and explain the failure of previously synthesized analogues of pironetin to improve potency. The new model for pironetin binding by α-tubulin enables the rational design of analogues directed to the creation of a new generation of anticancer drugs.

## Results

### Pironetin complex

Crystals of the α, β-tubulin heterodimer (T2), the stathmin-like protein RB3 (R) and tubulin tyrosine ligase (TTL) were prepared following published procedures^9^. This protein complex, denoted T2R-TTL, has been used in previous structural studies of tubulin-modifying agents. Its advantage is that it contains two heterodimers of tubulin and is thus a mimic for the biological polymer whilst being tractable in terms of solubility and structural rigidity^7^,^8^. We determined the structure of an apo (no ligand soaked) T2R-TTL complex crystal to 2.2-Å resolution to provide structure grown under identical conditions for comparison with the pironetin complex we obtained. The apo T2R-TTL complex is essentially identical to that previously described^9^,^10^,^11^, briefly two α, β-tubulin dimers are arranged as head to tail (denoted α1β1–α2β2; subunits A, B, C and D, respectively) with the long α-helix of the stathmin-like peptide (subunit E) contacting both tubulin dimers. TTL (subunit F) docks onto α1-tubulin (subunit A). Crystals soaked for longer than 16 h within 0.5 mM pironetin visibly deteriorated, gave rise to irregular diffraction spots with poor diffraction data at resolutions lower than 5 Å. Over 20 crystals soaked with pironetin for times ranging from 1 to 24 h were screened and one crystal soaked for 3 h gave high-quality data that we were able to process to a resolution of 2.6 Å (Table 1). The structures of apo T2R-TTL complex and T2R-TTL-pironetin complex are essentially identical. The two α-tubulin subunits (A and C) clearly have GTP bound. In β2-tubulin (subunit D) of both structures, the density is better fitted with GTP than GDP. In β1-tubulin (subunit B) of both structures, the density is more consistent with GDP but not unambiguously so (Supplementary Fig. 3). This nucleotide pattern has been previously reported^27^. Comparison of the residues that surround the nucleotide-binding site (by superposition) shows no difference between the subunits B and D in both structures. Thus, in our crystals either both B subunits do contain GTP or the change from GTP to GDP has occurred in the B subunit without any change in protein structure.

**Figure 2:**
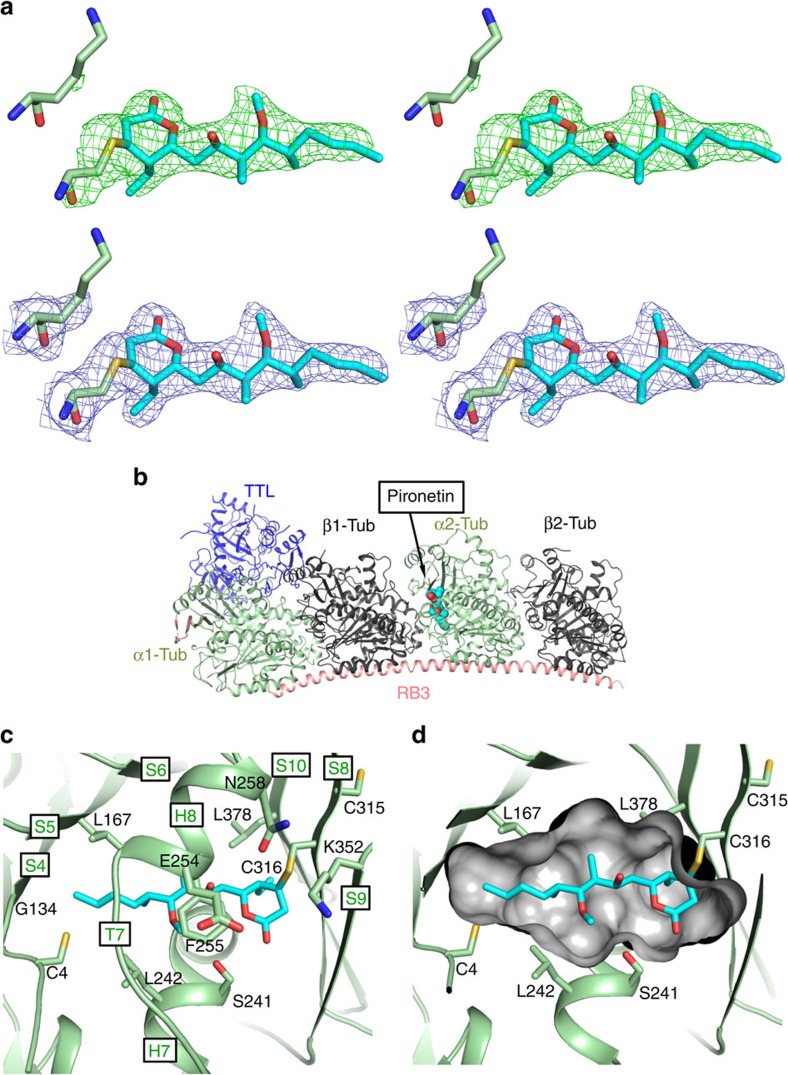
Structure of the tubulin-RB3-TTL-pironetin complex. (**a**) Stereo views of experimental Fo–Fc difference electron density contoured at 3*σ* in green for pironetin and the side chains of C316 and K352 and the final refined 2Fo–Fc electron density contoured at 1*σ* in blue. The final refined positions of pironetin, C316 and K352 are shown in stick with carbons coloured cyan for pironetin and green for protein, oxygens red and nitrogens blue. (**b**) Structure of tubulin-RB3-TTL in complex with pironetin in which pironetin is bound to α2-tubulin. The protein subunits are shown in cartoon. The ligands are shown as spheres with atoms coloured as in **a**. (**c**) Close-up view of pironetin in the pocket of α-tubulin monomer. (**d**) Cross-section view of the surface of the binding pocket of pironetin. K352 previously reported to be modified is shown alongside other residues discussed in the text.

The Fo–Fc difference electron density of the soaked crystals unambiguously identifies a covalent adduct at Cys316 of α2-tubulin (subunit C)(Fig. 2a). No additional clear density was identified around any other residue; we were particularly careful to check Lys352 (Fig. 2a and Supplementary Fig. 4). Pironetin was built into the positive Fo–Fc density with a good fit (Fig. 2a). The molecule was refined as a thiol Michael adduct using a dictionary from PRODRG server^28^. The binding site is entirely contained within α2-tubulin subunit (Fig. 2b). We did not model a second pironetin at Cys316 of α1-tubulin subunit, although we observed additional weak Fo–Fc density there. Cys316 is found on β-strand S8 (we have followed the published nomenclature of tubulin secondary structure elements^6^) and the pironetin molecule adopts an extended conformation that is perpendicular to the antiparallel β-sheet (strands S7, S8, S9 and S10) (Fig. 2c). The lactone ring of pironetin sits in a pocket formed by the residues from the antiparallel β-sheet (Fig. 2c,d). The ring and its ethyl substituent make van der Waal contacts with Leu378 and the main chain around Cys316. The hydroxyl alkyl chain extends into a channel that is formed by the helices H7, H8, β-strand S5 and loop T7 that connects H7 to H8 (Fig. 2c,d). The side chain of Phe255 (H8), Leu242 (H7) and Leu167 (S5) in particular makes van der Waal contacts with the chain (Fig. 2c). The terminal methyl group of pironetin sits in a large pocket formed by β-strands S1, S4 and S5 (part of another parallel β-sheet) and makes van der Waal contacts with Cys4, Gly134 and Leu167 (Fig. 2c,d).

### Mass spectrometry

**Figure 3:**
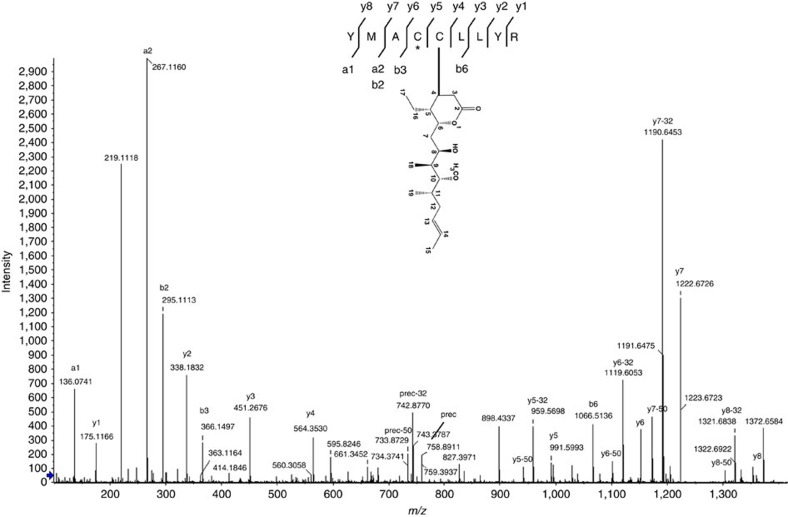
MS/MS fragmentation pattern for pironetin covalently modified peptide from α-tubulin .The spectrum clearly shows the covalent binding of pironetin to C316 rather than the adjacent C315, which was modified by carbamidomethyl (marked by *). The pironetin containing fragments show characteristic loses of 32 and 50 *m*/*z*. Prec denotes precursor ion.

We incubated α, β-tubulin with pironetin at a molar ratios of 1.1:1 and 1:20 (protein:pironetin) for 3 h at 20 °C. The reactions were quenched by addition of free cysteine, and the protein was separated on SDS–polyacrylamide gel electrophoresis (SDS–PAGE). The bands corresponding to α and β-tubulin were excised, subjected to reduction, alkylation and in-gel digestion with trypsin followed by analysis with nLC-ESI MS/MS. Under both treatments (1.1:1 and 1:20) we observed the pironetin covalently modified peptide (312-YMACCLLYR-320). Fragmentation of this peptide shows that pironetin is attached to Cys316 but not to Cys315. Detailed inspection of the MS/MS fragmentation pattern showed all the y ions containing Cys316 displayed diagnostic loses of 32 and 50 Da from pironetin (Fig. 3).

No pironetin binding was detected in observed peptides that contained Cys200, 213, 295, 347 and 376. Peptides containing Cys4, 20, 25, 129 and 305 were not observed so modification of these cannot be definitively excluded by mass spectrometry but clearly not any modification was found on those Cys residues in crystal structure (Supplementary Fig. 4). In the untreated protein we observed the 340-TIQFVDWCPTGFK-352 peptide. We were also able to see this (unmodified) peptide in the 20:1 pironetin-treated protein (Supplementary Fig. 5). Further, were the protein modified, the lysine would no longer be a trypsin cleavage site giving rise to the modified peptide 340-TIQFVDWCPTGFKVGINYEPPTVVPGGDLAK-370. We performed a product ion scan to specifically isolate the predicted *m*/*z* for this adduct but no signal was found.

### Structural changes on pironetin binding

**Figure 4:**
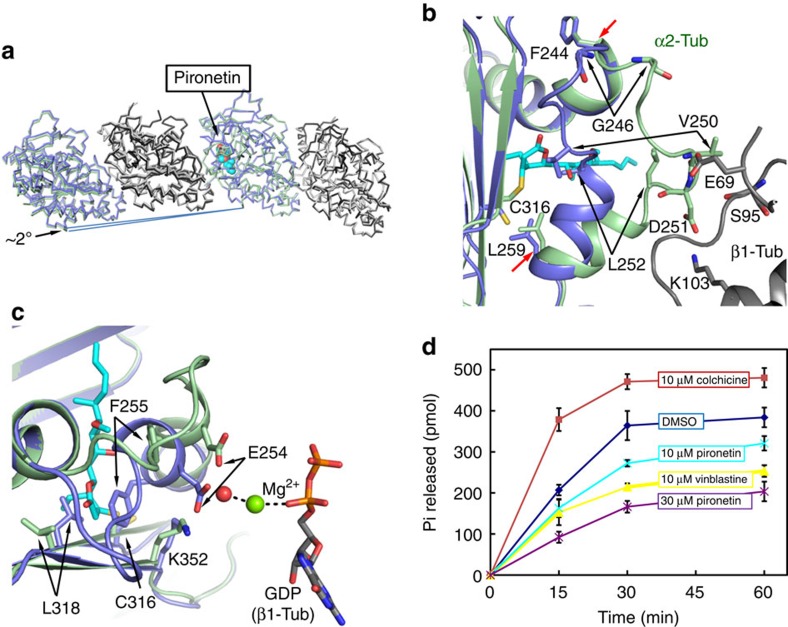
Changes in α-tubulin on pironetin binding. (**a**) Comparison of the structures of tubulins in apo form and in complex with pironetin. The tubulin dimers are represented as ribbons. In apo form, α-tubulin is coloured blue and β-tubulin light grey. In the complex with pironetin, α is green and β dark grey. (**b**) Large main chain changes between apo form (blue) and complex form (green) in H8 and the T7 loop, which links H7 and H8. Two red arrows at L259 and F244 mark the start and stop of the loop. G246, V250 and L252 are shown in sticks and marked with black arrows shift over 8 Å. (**c**) The catalytic residue E254 of α-tubulin is reoriented along with F255 on pironetin binding. (**d**) The GTPase activity of tubulin is reduced by pironetin similar to that observed with vinblastine and opposite to the stimulation seen with colchicine.

At a gross level, the binding of pironetin to α2-tubulin subunit has resulted in a 2° rotation (2.2° between our structures and 1.9° when compared with the existing apo structure (PDB code: 4I55)) in the relative positions of the α1β1 heterodimer with respect to the α2β2 heterodimer (Fig. 4a). At the secondary structural level there is a pronounced shift in the position of loop T7 (residues Phe244 to Leu259): the Cα atom of Val250 has moved over 11 Å, whilst Leu252 has shifted over 6 Å and Gly246 by 8 Å (Fig. 4b). The movement is a direct consequence of pironetin binding, as in the apo structure this part of the T7 loop occupies the same volume as the pironetin hydroxyl alkyl chain. In the complex, the T7 loop is stabilized by a contact consistent with a salt bridge between Asp251 (from α2-tubulin, subunit C) and Lys103 (from β1-tubulin, subunit B; Fig. 4b). There are further interactions consistent with hydrogen bond interactions between the amides of Val250 in α2-tubulin with the side chain of Glu69 from β1-tubulin (Fig. 4b). The residues on either side of Cys316 on strand S8 undergo small shifts with different side chain conformers for both Cys316 and Leu318 that are required for pironetin binding (Fig. 4c). In addition, there are small changes in the positions of atoms on the neighbouring strands.

There is also a shift in the position of the N-terminal end of helix H8 that correlates with the changes in the T7 loop, notably the side chain of Phe255 has undergone a large re-orientation (Fig. 4c). Glu254 is a key catalytic residue that in the apo structure binds to water molecules that in turn binding to a Mg^2+^ ion of the neighbouring β1-tubulin (Fig. 4c). The Mg^2+^ ion is in contact with α-phosphate of GDP. However, Glu254 in this complex lacks the link to Mg^2+^ due to a movement of 4 Å of the carboxylate group. Glu254 has been proposed to enhance the tubulin GTPase activity^29^,^30^. The distortion of the precise position of Glu254 and the resulting lower catalytic capacity for GTPase activity of β-tubulin (Fig. 4d) may be an additional mechanism of pironetin to destabilize the assembly of tubulin into microtubules.

Notably, the changes in the structure of α-tubulin are focused at the inter-dimer interface between α2-tubulin (subunit C) and β1-tubulin (subunit B). It is this interface between heterodimers that directly and exquisitely controls tubulin polymerization^7^,^8^,^10^. Electron micrographs and sedimentation analysis were used to confirm that the tubulin and pironetin used in this study showed the expected disruption of microtubule formation (Supplementary Fig. 6). Controls of tubulin treatment with colchicine and vinblastine gave the expected results^8^,^31^ (Supplementary Fig. 6). We take these results to confirm the authenticity of both the protein and the compound used.

## Discussion

Mass spectrometric and crystallographic data establish that pironetin binds covalently to Cys316 of α-tubulin. The mass spectrometric analysis was carried out on tubulin heterodimer in solution (without the other proteins used to obtain crystals) to ensure the modification of Cys316 was not the result of crystal packing or an artefact of T2R-TTL protein complex used. Both techniques identify that pironetin binding is specific, in that other cysteines on the surface of the protein, available for reaction, were not modified. Unfolding the protein (during mass spectrometric analysis) in the presence of excess pironetin does result in additional nonspecific cysteine modification. This indicates that the specific Cys316 modification seen for the folded protein requires recognition of pironetin, consistent with the crystal structure which identifies a binding pocket in the protein. Chemical considerations suggest that the thiol of cysteine will be a more potent nucleophile than amine of lysine for Michael addition. Thus, cysteine modification by the α/β unsaturated lactone of pironetin might be expected, these results contradict an earlier study that had reported covalent binding to Lys352 (ref. 22). The identification of Lys352 as the target had relied on an Ala mutational scan of lysine and cysteine residues from Lys280 to Lys352 following treatment with a biotinylated derivative of pironetin^22^. The data showed that the mutations K352A and N258A caused large reduction of binding of biotin-modified pironetin whilst mutations C315A and C316A slowed down but not reduce the overall amount of binding to the biotinylated derivative^22^. Interestingly, this study showed that pironetin binding could be reversed under relatively mild conditions, at pHs below 4.5 or above 8.5 (ref. 22). We suggest this observation is more consistent with a thiol adduct of pironetin rather than the more chemically stable amine adduct. We examined the residual density in the crystal structure at Lys352 (Supplementary Fig. 4) but see no density for any modification. Mass spectrometry of the pironetin-treated protein shows no evidence for any modification on Lys352. In fact, mass spectrometry detects the unmodifed Lys352 ((Supplementary Fig. 5) even after treatment of the protein with high ratio of pironetin, this is positive evidence that Lys352 is not labelled by native pironetin.

The structure of tubulin complexed to pironetin shows that the binding pocket is entirely contained in α-tubulin subunit close to the interface between different tubulin heterodimers (such as α2-tubulin and β1-tubulin subunit). This pocket absent in the apo structure and is created by an induced-fit mechanism with a large re-orientation of T7 loop. Such induced-fit binding sites are rarely predicted by modelling highlighting the importance of experimental structural study. The previous study had used biotinylated pironetin (Supplementary Fig. 2), and we note the pocket seen here could not accommodate biotinylated pironetin as the biotin component would clash with Phe255 and other residues in the pocket. As we are unable to synthesize or source the biotinylated analogue of pironetin, we have not been to investigate the behaviour of the biotinylated pironetin using our analytical approaches.

The disappointing results from the design and synthesis of pironetin analogues^24^,^25^ can now be rationalized in light of the new structural data. The loss of the ethyl substituent on the lactone ring disrupts the hydrophobic interactions with Leu378 and would create an unfavourable void. The most potent analogue of pironetin preserved this group but missed two methyl substituents from the alkyl chain. These methyl groups do not make direct interactions but favour the extended conformation of pironetin and this, we suggest, is critical in inducing the interaction between Phe255 and the drug.

In the crystal structure, pironetin is modelled bound to one of two α-tubulin molecules (α2-tubulin, subunit C), there is only weak density for the molecule in α1-tubulin (subunit A) and disordering of some neighbouring residues. We suggest the reason for this asymmetric binding of pironetin is that the stathmin-like peptide RB3, necessary for crystallization of the complex, has extensive interactions with strand S9 as well as loop T7 of α-tubulin. We hypothesize that modification of the α1-tubulin subunit (subunit A) would disrupt RB3-(α-tubulin) interactions and thus crystal packing, consistent with the poor quality of diffraction for those crystals with prolonged soaking times.

**Figure 5:**
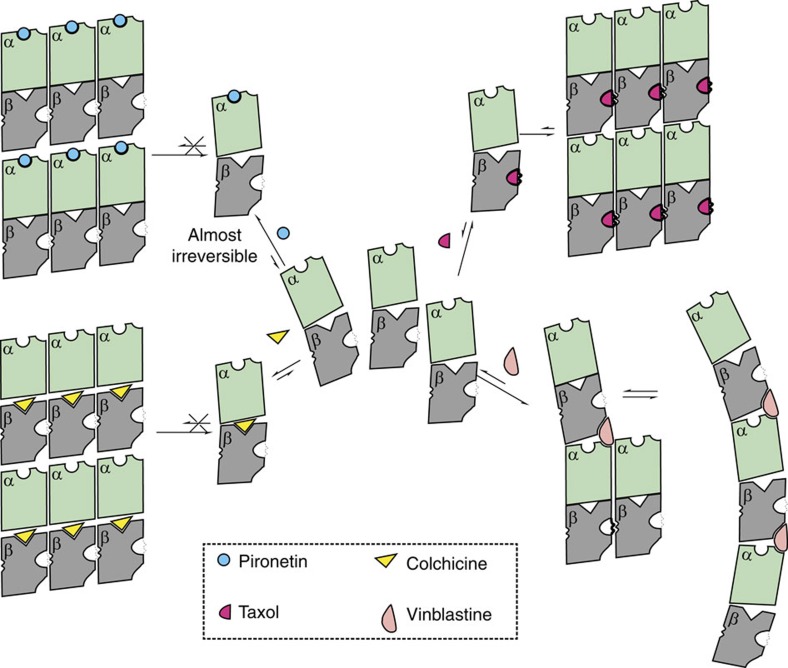
The molecular mechanism of pironetin and other tubulin-targeting agents to alter the tubulin-microtubule dynamics. The tubulin-targeting agents colchicine, vinblastine and taxol are shown. This figure is inspired by a previous publication^10^ but designed and developed with the data in this study.

Since pironetin perturbs the longitudinal contacts between the tubulin heterodimers, we suggest that this is the basis by which it promotes the disassembly of microtubules (Fig. 5). The vinca class of alkaloids, such as vinblastine, bind to a site on β-tubulin, which is also at the interface between heterodimers and act by perturbing the polymerization^8^. Interestingly, vinblastine in the crystal structure of its complex with tubulin results in a change in the position of the key catalytic Glu254 of α-tubulin^8^, the same residue undergoes a shift, but a different one, in the pironetin complex. This overlap in structural effects may underpin the observed competition between vinblastine and pironetin^21^. Colchicine binds to β-tubulin at intra-dimer interface (the opposite end of β-tubulin than the vinca alkaloids)^7^ also inhibits polymerization. Taxol in contrast, binds at a third site in the middle of β-tubulin and stabilizes the flexible M loop promoting stabilization of microtubules^6^,^9^.

Pironetin has been shown to possess antitumour activity and is the only known compound that modifies microtubule assembly by covalent modification of α-tubulin. Thus, pironetin-like compounds have attractive potential as treatments of tumours in which switch in the isoform of β-tubulin has rendered the cancer resistant to β-tubulin-binding drugs, including taxol. The covalent attachment of pironetin to tubulin all but eliminates the vulnerability to efflux pumps. The complex structure of pironetin and tubulin reveals a previously unknown but highly specific ligand binding site. Exploitation of this binding site can be now be used to create a new class of anti-cancer compounds targeting α-tubulin.

## Methods

### Materials

Clones of stathmin-like domain of RB3 (RB3-SLD) and TTL were obtained as kind gifts from Dr Benoît Gigant (CNRS, France) and Dr Michel O. Steinmetz (PSI, Switzerland). Pironetin was purchased from Biomol or Oskar Tropitzsch; colchicine, Bis Tris Propane, tyrosine, AMPPCP (adenylylmethylenediphosphonate or β, γ-methyleneadenosine 5′-triphosphate) and DTT (dithiothreitol) from Sigma-Aldrich and vinblastine from Selleck. Other frequently used reagents were purchased from Sigma-Aldrich or similar quality suppliers. A single batch of porcine brain tubulin (>99% pure, Catalogue No: T238P) was sourced from Cytoskeleton Inc., snap frozen immediately on delivery into 10 mg ml^−1^ aliquots and stored at –70 °C until required.

### Methods

*Protein expression and purification*. On the basis of a published procedure^9^, RB3-SLD was expressed in and purified by anion-exchange chromatography from *Escherichia coli*, followed by a final step of gel filtration in 10 mM HEPES-NaOH buffer (pH 7.2), 150 mM NaCl and 2 mM DTT. Pure RB3-SLD protein was concentrated to 10 mg ml^−1^ and stored at −80 °C. His-tagged TTL was purified from *E. coli* by Ni^2+^ affinity chromatography, followed by gel filtration in 20 mM Bis Tris Propane buffer (pH 6.5), 200 mM NaCl, 2.5 mM MgCl_2_, 5 mM β-mercaptoethanol, 1% glycerol and finally concentrated to 20 mg ml^−1^ and stored at −80 °C. The purity of RB3 and TTL were assessed by SDS–PAGE. Porcine brain tubulin was purchased and supplied at 10 mg ml^−1^ in GTB buffer (general tubulin buffer: 80 mM PIPES (pH 6.9); 2 mM MgCl_2_; 0.5 mM EGTA; and 1 mM GTP) and preserved at −80 °C until use.

*Structural biology*. A protein solution consist of tubulin (10 mg ml^−1^), TTL (20 mg ml^−1^) and RB3 (10 mg ml^−1^), with molar ratios of each component at 2:1.3:1.2 (Tubulin:RB3:TTL), was incubated on ice. To this was added 1 mM AMPPCP, 5 mM tyrosine and 10 mM DTT, and the resulting mixture concentrated to 20 mg ml^−1^ at 4 °C. Sitting-drop crystallization drops contained 1 μl protein solution and 1 μl of 6% PEG 4000, 5% glycerol, 0.1 M MES, 30 mM CaCl_2_ and 30 mM MgCl_2_, pH 6.7 at 20 °C. Crystal growth was accelerated by seeding^27^, and crystals appeared after incubation at 20 °C in the same day and reached a length of 200–300 μm within 3–5 days.

A volume of 0.1 μl of pironetin, dissolved in dimethylsulphoxide at 10 mM concentration, was added to a sitting drop containing crystals at 20 °C. The time for soaking was varied between 1 and 24 h, with the crystals examined for visibly cracking or degradation. Crystals (both native and soaked) were removed from the drop on a loop dipped into cryoprotectant (8% PEG 4000, 30 mM MgCl_2_, 30 mM CaCl_2_, 0.1M MES, pH6.7 and 20% glycerol) before being flash-cooled in liquid nitrogen.

X-ray diffraction data were collected at beamline BL19U1 at Shanghai Synchrotron Radiation Facility (SSRF, National Center for Protein Science Shanghai, Institute of Biochemistry and Cell Biology, Chinese Academy of Sciences, P. R. China) using the MX225 CCD (charge-coupled device) detector at a wavelength of 0.97853 Å. Data were indexed, integrated and scaled using Xia2 package^32^,^33^,^34^,^35^. The structures were solved by the molecular replacement method, with an existing apo structure T2R-TTL (no soaking ligands) (PDB code: 4I55) as the search model by using Phaser^36^. Manual model building and following refinement were done by using Coot^37^ and Refmac5 (ref. 38). The restraint library for pironetin was produced from PRODRG server^28^. The final data collection and refinement statistics are summarized in Table 1.

*Mass spectrometry*. We incubated 20 μl α, β-tubulin at 27 μM concentration with inadequate and excessive pironetin at a molar ratios of 1.1:1 and 1:20 (protein:pironetin) at 20 °C for 3 h quenched by free amino acid cysteine and then ran the reactions on SDS–PAGE. The excess free amino acid cysteine is crucial to mask the residual pironetin in the sample before denaturing tubulin for SDS–PAGE. If excess pironetin was not quenched before unfolding on SDS–PAGE, additional cysteine residues (but not lysine) were modified. The gel band was excised and cut into 1-mm cubes. These were then subjected to in-gel digestion, using a ProGest Investigator in-gel digestion robot (Genomic Solutions) following the standard protocols^39^. Briefly the gel cubes were destained by washing with acetonitrile and subjected to reduction, with dithiothreitol, and alkylation, with iodoacetamide, before digestion with trypsin at 37 °C in 25 mM ammonium bicarbonate buffer. The peptides were extracted with 5% formic acid and concentrated down to 20 μl using a SpeedVac (Thermo Savant).

The peptides were then separated on an Acclaim PepMap 100 C18 trap and an Acclaim PepMap RSLC C18 column (Thermo Fisher Scientific), using a nanoLC Ultra 2D plus loading pump and nanoLC as-2 autosampler (Eksigent). The peptides were eluted with a gradient of increasing acetonitrile, containing 0.1% formic acid (5–40% acetonitrile in 16 min, 40–95% in a further 1 min, followed by 95% acetonitrile to clean the column, before re-equilibration to 5% acetonitrile). The eluant was sprayed into a Triple TOF 5600^+^ electrospray tandem mass spectrometer (Sciex) and analysed in information-dependent acquisition mode, performing cycles of 250 ms of MS followed by 100 ms MS/MS analyses on the 15 most intense peaks seen by MS.

The MS/MS data file generated via the ‘Create mgf file' script in PeakView (Sciex) was analysed using the Mascot algorithm (Matrix Science), against an in-house database to which α-tubulin subunit sequence had been added, with trypsin as the cleavage enzyme and carbamidomethyl modification of cysteines, pironetin modification of cysteines, pironetin modification of lysines, methionine oxidation and deamidation of glutamines and asparagines as a variable modifications.

A repeat injection was made using the same HPLC conditions with the mass spectrometer operating in product ion scan mode to specifically isolate and characterize the *m*/*z*'s for fragmentation of 312-YMACCLLYR-320 and 340-TIQFVDWCPTGFKVGINYEPPTVVPGGDLAK-370 with pironetin adducts and carbamidomethyl adducts covalently bound.

*Transmission electron microscopy*. Tubulin (10 μM) solution was prepared in GTB-glycerol buffer (80 mM PIPES buffer (pH 6.9), 2 mM MgCl_2_, 0.5 mM EGTA, 1 mM GTP and 10% glycerol) at 4 °C. Tubulin in this buffer does not assemble at 4 °C but will assemble spontaneously at 37 °C. Different microtubule-destabilizing agents colchicine, vinblastine and pironetin were added to tubulin at 10 μM and incubated at 37 °C for 30 min, respectively. A volume of 5 μl of sample was added to a 230-mesh per inch formvar, supported by carbon films, adsorbed for 2 min, washed twice with water and negatively stained for 60 s with 2% (w/v) phosphotungstic acid. A FEI T12 transmission electron microscope was used for observation at 80 Kv, and the pictures were recorded into a Serial EM software by a 2k × 2k Gatan CCD camera

*Tubulin sedimentation analysis*. Tubulin (20 μM) solution was prepared in GTB-glycerol buffer. Different microtubule-destabilizing agents at indicated concentrations were added to the tubulin and incubated at 37 °C for 30 min. Ultracentrifugation (250,000*g*, 15 min) was performed to separate the pellets (aggregates of tubulin) and supernatants (tubulin), which were both subjected to SDS–PAGE, respectively, and stained by Coomassie brilliant blue.

*GTPase assay*. Analysis of tubulin GTPase activity was performed using the QuantiChromTM ATPase/GTPase Kit (DATG-200) from BioAssay Systems following the manufacturer's instruction and the published GTPase assay of tubulin^40^,^41^. The final volume of the GTPase assay is 300 μl and is carried out in GTB buffer with 10 μM of the assay compounds (colchicine, vinblastine and pironetin, all dissolved in dimethylsulphoxide at 0.5 mM concentration (50-fold stock solution)) and is initiated by addition of 66 μl of tubulin (giving a final concentration of tubulin of 20 μM). At different time intervals (0, 15, 30 and 60 min), 70 μl reaction solution was removed and rapidly mixed with perchloric acid (final concentration of 2.5%) to stop the reaction. Protein was removed from the reaction by ultrafiltration. A volume of 200 μl of the developing reagent (supplied in the kit) was added to 40 μl reaction filtrate and incubated for 30 min at room temperature. The absorbance at 620 nm was measured by a Spectramax M5 Microtiter Plate Luminometer (Molecular Devices, USA) and the data were plotted to show the activity change on the microtubule-destabilizing agents with the phosphate concentration released at 0 min as the blank.

### Data availability

The coordinates and structure factors that support the finding of this study have been deposited in the Protein Data Bank with the accession codes 5JQG and 5FNV, respectively, (http://www.rcsb.org/pdb/home/home.do). Further data of transmission electron microscopy, tubulin sedimentation analysis and GTPase assay are available from the corresponding author L.C. (ljchen@scu.edu.cn) and others are available from X.Z. (zhuxiaofeng@scu.edu.cn) on request.

## Additional information

**How to cite this article**: Yang, J. *et al.* Pironetin reacts covalently with Cysteine-316 of a-tubulin to destabilize microtubule. *Nat. Commun.* 7:12103 doi: 10.1038/ncomms12103 (2016).

## Supplementary Material

Supplementary Information Supplementary Figures 1-6 and Supplementary References.

## Figures and Tables

**Figure 1:**
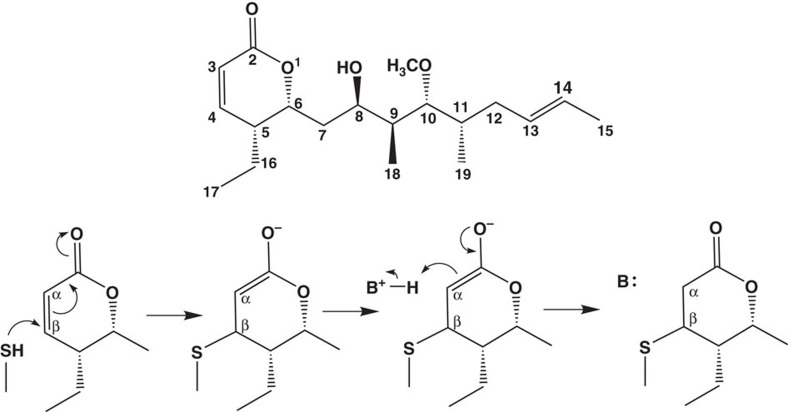
Chemical structure of pironetin and the reaction scheme with cysteine. Cysteine as a thiol nucleophile undergoes a Michael-type addition to pironetin, in which a protonated base (denoted as B^+^) or solvent accepts the electron from enolate intermediate.

**Table 1:** Data collection and refinement statistics.

	**Apo T2R-TTL (5JQG)**	**T2R-TTL-pironetin (5FNV)**
*Data collection*		
Space group	*P*2_1_2_1_2_1_	*P*2_1_2_1_2_1_
Cell dimensions		
*a*, *b*, *c* (Å)	105.05, 158.12, 180.63	106.16, 157.20, 181.68
*α*, *β*, *γ* (°)	90, 90, 90	90, 90, 90
Resolution (Å)	35.22–2.24 (2.30–2.24)	45.5–2.61 (2.68–2.61)*
*R*_merge_	0.074 (0.574)	0.058 (0.491)
*I*/*σI*	14.9 (2.8)	15.8 (1.6)
Completeness (%)	100 (99.9)	94.8 (67.5)
Redundancy	6.7 (6.3)	5.3 (2.5)
CC (1/2)	0.998 (0.846)	0.999 (0.652)
		
*Refinement*		
Resolution (Å)	35.22–2.24	41.76–2.61
No. of reflections	144,634	87,988
*R*_work_/*R*_free_	0.181/0.212	0.186/0.226
No. of atoms	17,646	17,276
Protein	17,126	16,999
Ligand/ion	187	209
Water	333	68
*B*-factors	49.4	80.5
Protein	49.7	80.7
Ligand/ion	43.5	69.8
Water	39.3	54.8
R.m.s. deviations		
Bond lengths (Å)	0.015	0.012
Bond angles (°)	1.68	1.64

R.m.s., root mean squared.

The resolution limits were determined by half-data set correlation (CC (1/2))^42^ and values in parentheses are for highest-resolution shell.

^*^One crystal was used for data collection and structure determination.
