# Supplementary material for: Pironetin reacts covalently with cysteine-316 of α-tubulin to destabilize microtubule
Source: Nat Commun. 2016 Jun 30;7:12103. doi: 10.1038/ncomms12103 (PMC4931326; doi:10.1038/ncomms12103)
Supplement: Supplementary Information — Supplementary Figures 1-6 and Supplementary References. [file ncomms12103-s1.pdf]

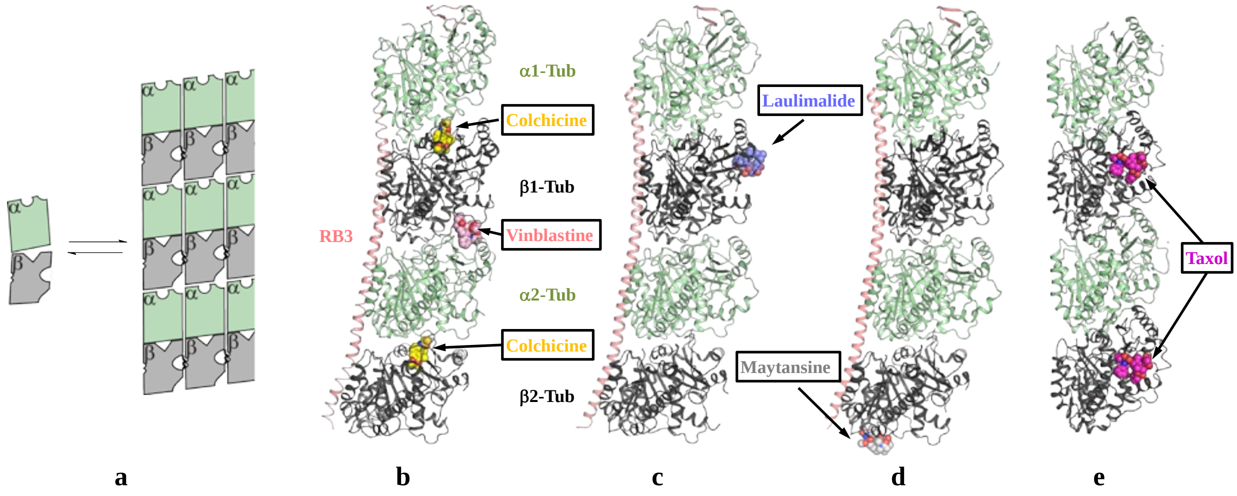

Supplementary Figure 1 Structures of microtubule and tubulin (**a**)The cartoon of head to tail arrangement of tubulin dimers to form microtubules and undergo a curved-to-straight conformational transition with longitudinal and lateral contacts between dimers; (**b-e**) Structures of tubulin showing the binding site of current drugs: (**b**) Vinblastine and colchicine which inhibit polymerization binds within the β subunit[1]; (**c**) Laulimalide which also binds to the β subunit inhibits depolymerization of microtubules[2]; (**d**) Maytansine which inhibits polymerization binds to the β subunit[3]; (**e**) Taxol which binds to the β subunit inhibits depolymerization of microtubules[4]. The protein subunits are shown in cartoon. The ligands are shown as spheres with oxygen atoms are colored red, nitrogens blue and the carbons in colchicine, vinblastine, laulimalide, maytansine and taxol are coloured in yellow, pink, blue, white and purple respectively.

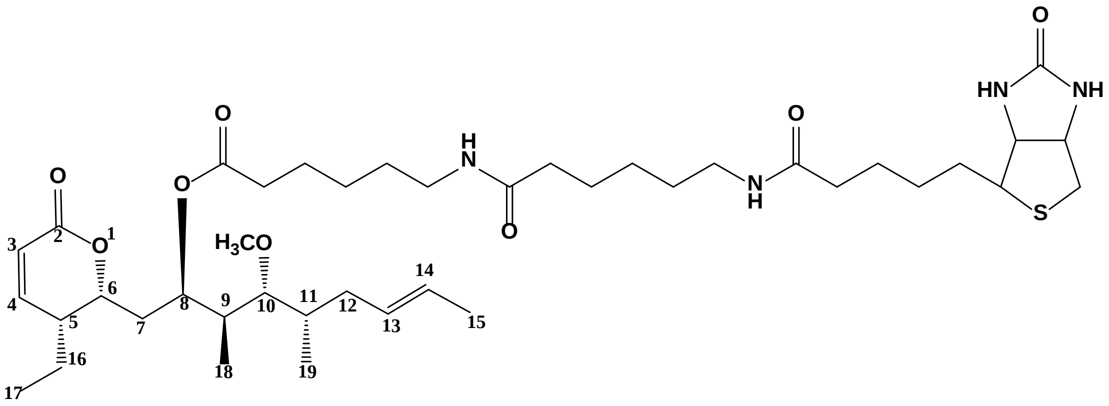

Supplementary Figure 2 Chemical structure of biotinylated pironetin[5].

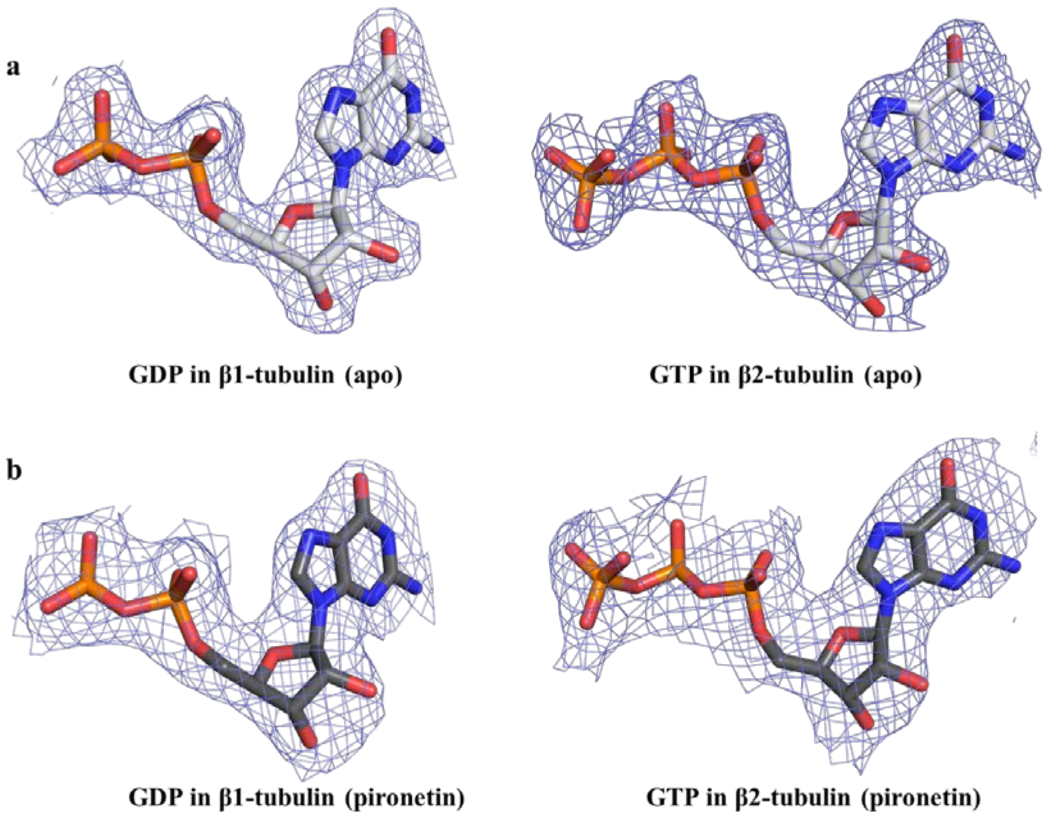

Supplementary Figure 3 The final refined 2Fo-Fc electron density contoured at 1σ for nucleotide in β1 and β2-tubulin subunits of apo T2R-TTL structure (a) and of T2R-TTL-pironetin complex structure (b). Nucleotide molecules in tubulin are shown in stick with oxygens red, nitrogens blue and carbons colored light grey and dark grey in apo and pironetin complex respectively.

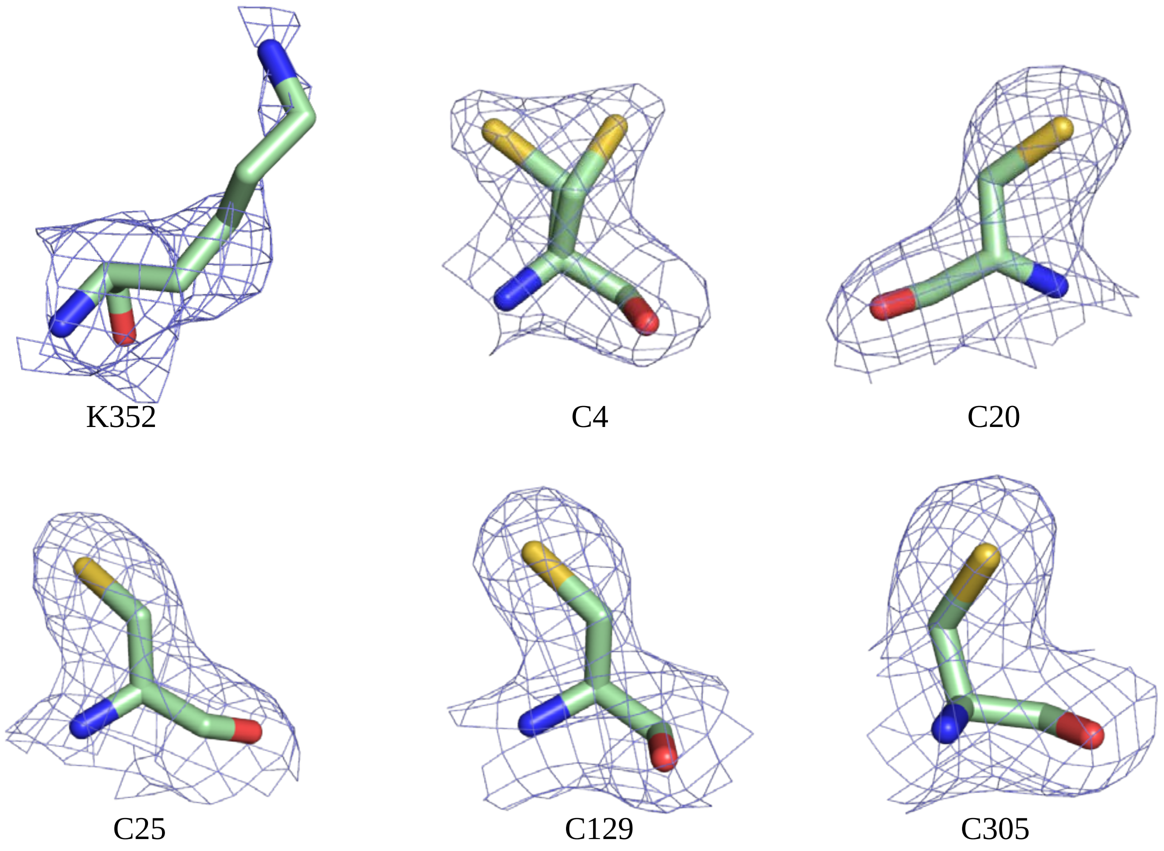

Supplementary Figure 4 The final refined 2Fo-Fc electron density for Lys352 (contoured at 0.067e Å^{-3} (0.6 σ)) and Cys residues in C subunit (contoured at 0.14e Å^{-3} (1 σ)) which were not detected in mass spectrometry studies including Cys4, Cys20, Cys25, Cys129 and Cys305. Protein residues are shown in stick with carbons colored green, oxygens red and nitrogens blue.

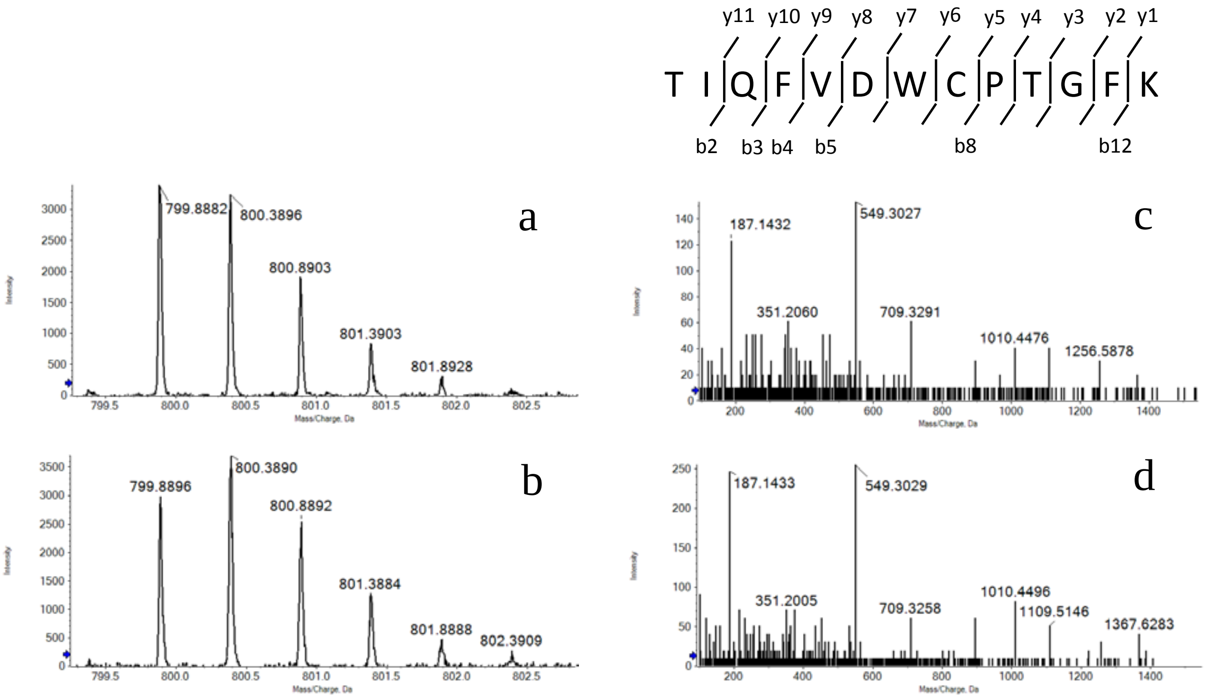

Supplementary Figure 5 MS and MS/MS spectra for peptide 340-TIQFVDWCPTGFK-352 from α-tubulin. The presence of this peptide in the trypsin digest of both a control sample, which had not been incubated with pironetin, and a sample that had been incubated with pironetin demonstrates that K352 does not react with pironetin. **(a)** MS spectrum for a peptide corresponding to the predicted mass for 340-TIQFVDWCPTGFK-352 in a trypsin digest of α-tubulin which had not been incubated with pironetin; **(b)** corresponding MS spectrum from a trypsin digest of α-tubulin which had been incubated with pironetin; **(c)** MS/MS fragmentation pattern for 799.89 m/z from the trypsin digest of α-tubulin which had not been incubated with pironetin confirming this m/z as peptide 340-TIQFVDWCPTGFK-352 from α-tubulin; **(d)** MS/MS fragmentation pattern for 799.89 m/z from the trypsin digest of α-tubulin which had been incubated with pironetin, confirming this m/z as peptide 340-TIQFVDWCPTGFK-352 from α-tubulin.

a

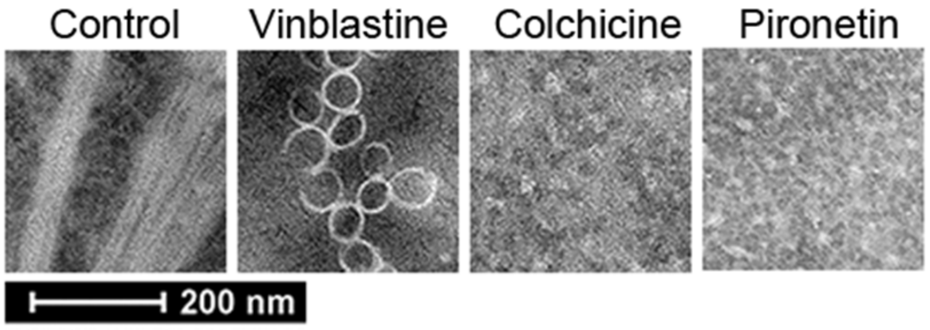

b

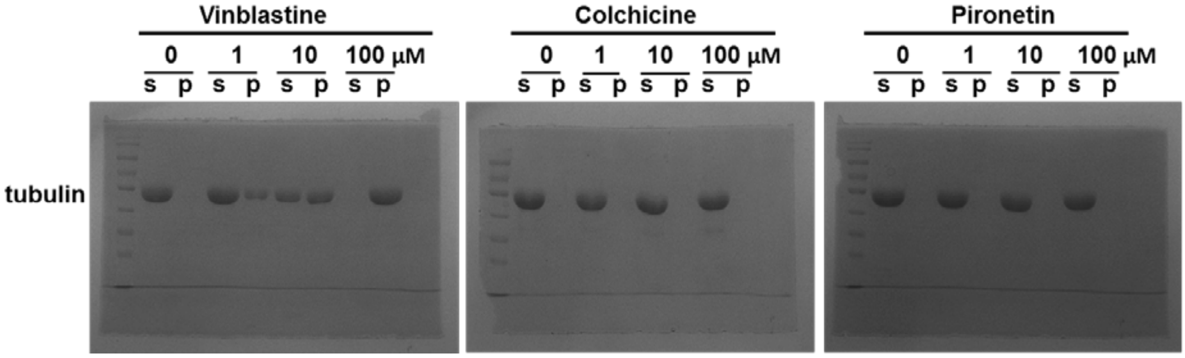

Supplementary Figure 6 Biological effect of pironetin in microtubule dynamics along with the tubulin-targeting agents vinblastine and colchicine. (**a**) Electron micrograph of negatively stained tubulin sample treated with vinblastine, colchicine and pironetin. Scale bar, 200 nm; (**b**) Variation in aggregation behavior of tubulin samples was assayed by analysing the tubulin content in the supernatant (S) and pellet (P) after centrifugation of mixtures with tubulin-targeting agents at the indicated concentrations. These results of vinblastine[1]and colchicine[6] are as previously reported and confirm the protein and pironetin used here are authentic.

Reference for supplementary materials

1. Gigant, B. et al. Structural basis for the regulation of tubulin by vinblastine. *Nature* **435**, 519-22 (2005).
2. Prota, A.E. et al. Structural basis of microtubule stabilization by laulimalide and peloruside A. *Angew Chem Int Ed Engl* **53**, 1621-5 (2014).
3. Prota, A.E. et al. A new tubulin-binding site and pharmacophore for microtubule-destabilizing anticancer drugs. *Proc Natl Acad Sci U S A* **111**, 13817-21 (2014).
4. Lowe, J., Li, H., Downing, K.H. & Nogales, E. Refined structure of alpha beta-tubulin at 3.5 A resolution. *J Mol Biol* **313**, 1045-57 (2001).
5. Usui, T. et al. The anticancer natural product pironetin selectively targets Lys352 of alpha-tubulin. *Chem Biol* **11**, 799-806 (2004).
6. Prota, A.E. et al. The novel microtubule-destabilizing drug BAL27862 binds to the colchicine site of tubulin with distinct effects on microtubule organization. *J Mol Biol* **426**, 1848-60 (2014).
